# Effects of jaw clenching on dynamic reactive balance task performance after 1-week of jaw clenching training

**DOI:** 10.3389/fneur.2023.1140712

**Published:** 2023-06-23

**Authors:** Cagla Fadillioglu, Lisa Kanus, Felix Möhler, Steffen Ringhof, Daniel Hellmann, Thorsten Stein

**Affiliations:** ^1^BioMotion Center, Institute of Sports and Sports Science, Karlsruhe Institute of Technology (KIT), Karlsruhe, Germany; ^2^Department of Prosthodontics, University of Würzburg, Würzburg, Germany; ^3^Department of Sport and Sport Science, University of Freiburg, Freiburg im Breisgau, Germany; ^4^Department of Diagnostic and Interventional Radiology, University Medical Center Freiburg, Faculty of Medicine, University of Freiburg, Freiburg im Breisgau, Germany; ^5^Dental Academy for Continuing Professional Development, Karlsruhe, Germany

**Keywords:** stomatognathic activity, postural control, temporomandibular joint, Posturomed, reflex phases, dual task

## Abstract

**Introduction:**

Good balance is essential for human daily life as it may help to improve the quality of life and reduce the risk of falls and associated injuries. The influence of jaw clenching on balance control has been shown under static and dynamic conditions. Nevertheless, it has not yet been investigated whether the effects are mainly associated with the dual-task situation or are caused by jaw clenching itself. Therefore, this study investigated the effects of jaw clenching on dynamic reactive balance task performance prior to and after 1 week of jaw clenching training. It was hypothesized that jaw clenching has stabilizing effects resulting in a better dynamic reactive balance performance, and these effects are not related to dual-task benefits.

**Methods:**

A total of 48 physically active and healthy adults (20 women and 28 men) were distributed into three groups, one habitual control group (HAB) and two jaw clenching groups (JAW and INT) that had to clench their jaws during the balance tasks at T1 and T2. One of those two groups, the INT group, additionally practiced the jaw clenching task for 1 week, making it familiar and implicit at T2. The HAB group did not receive any instruction regarding jaw clenching condition. Dynamic reactive balance was assessed using an oscillating platform perturbed in one of four directions in a randomized order. Kinematic and electromyographic (EMG) data were collected using a 3D motion capture system and a wireless EMG system, respectively. Dynamic reactive balance was operationalized by the damping ratio. Furthermore, the range of motion of the center of mass (CoM) in perturbation direction (RoM_CoM_AP_ or RoM_CoM_ML_), as well as the velocity of CoM (*V*_CoM_) in 3D, were analyzed. The mean activity of the muscles relevant to the perturbation direction was calculated to investigate reflex activities.

**Results:**

The results revealed that jaw clenching had no significant effects on dynamic reactive balance performance or CoM kinematics in any of these three groups, and the automation of jaw clenching in the INT group did not result in a significant change either. However, high learning effects, as revealed by the higher damping ratio values and lower *V*_CoM_ at T2, were detected for the dynamic reactive balance task even without any deliberate balance training in the intervention phase. In the case of backward perturbation of the platform, the soleus activity in a short latency response phase increased for the JAW group, whereas it decreased for HAB and INT after the intervention. In the case of forward acceleration of the platform, JAW and INT showed a higher tibialis anterior muscle activity level in the medium latency response phase compared to HAB at T1.

**Discussion:**

Based on these findings, it can be suggested that jaw clenching may lead to some changes in reflex activities. However, the effects are limited to anterior–posterior perturbations of the platform. Nevertheless, high learning effects may have overall overweighed the effects related to jaw clenching. Further studies with balance tasks leading to less learning effects are needed to understand the altered adaptations to a dynamic reactive balance task related to simultaneous jaw clenching. Analysis of muscle coordination (e.g., muscle synergies), instead of individual muscles, as well as other experimental designs in which the information from other sources are reduced (e.g., closed eyes), may also help to reveal jaw clenching effects.

## Introduction

Balance is one of the essential aspects of postural control and is crucial to accomplish daily routine activities, such as unassisted standing and walking. Impaired balance control may lead to an increased risk of falls and a reduced quality of life (1, 2). From a mechanical point of view, balance involves controlling the center of mass (CoM) with respect to the base of support (1). During standing, the CoM sways steadily within the body's base of support (i.e., static steady balance), whereas during perturbations, stability needs to be recovered to bring the CoM back to the allowed limits necessary for maintaining posture (i.e., dynamic reactive balance); (3). Given the importance of balance (1), it is valuable to improve its control mechanisms through balance training. This is recommended for performance enhancement in sports (4) to prevent injuries (5) and to decrease falls in at-risk groups (6, 7).

An important prerequisite for balance is the sensory input that derives from the somatosensory, visual, and vestibular systems and provides the central nervous system (CNS) with information regarding the state of the body and the environment. This sensory information is weighted in a task-dependent manner (8). For example, when the support surface is rapidly displaced (i.e., the dynamic reactive balance control is challenged), the CNS mostly relies on somatosensory inputs since these enable faster reactions than other systems of sensory input (1). Given the importance of somatosensory information for dynamic reactive balance control, any alteration that improves dynamic stability may be relevant for fall prevention, especially in unexpected external perturbations (2, 9).

A growing body of literature suggests that there is a close relationship between the stomatognathic system and balance (10–18). The underlying mechanisms have not yet been fully understood; however, in various studies (19–22), it was shown that jaw clenching like the Jendrassik maneuver (23) may lead to increased motor excitability and increased H-reflex responses. In addition, co-contraction behavior of the masticatory and neck muscles occurring as a result of complex neurophysiological interactions (24) may also contribute to improved postural control, for example, via a more stable head or gaze position (25–27). These results are neuroanatomically supported by findings in animal models which found neuronal links of the trigeminal nerve to numerous brainstem nuclei and all levels of the spinal cord (28).

Although jaw clenching has been shown to affect balance performance under both static (12, 29, 30) and dynamic conditions (10, 14), it is still unknown whether these effects are associated with the dual-task situation [i.e., influences of simultaneously performed additional motor tasks (31, 32)] or those specifically connected to jaw clenching. In general, when two tasks are performed simultaneously, performance decreases in one or both tasks (33), which can be explained by the limited capacity of attention (34). However, with respect to balance control, previous studies showed that combining a secondary task with a balance task may actually improve performance compared to a single-task condition (32). This phenomenon can be explained by altered attention and increased automatization of balance control processes (31, 35). Therefore, one might argue that stabilizing effects on balance control could be caused by the secondary task of jaw clenching.

To sum up, the acute positive effects of jaw clenching have been shown in various studies (10, 12, 14, 29, 30); however, it has not yet been evaluated if these effects are associated with dual-task benefits or specifically based on neurophysiological effects caused by jaw clenching. Therefore, this study established an intervention group (INT) that trained jaw clenching, so that it becomes an implicit task. The comparison with a group (JAW) that was only instructed in jaw clenching shortly before T1 and T2 and with a group without any training as well as instruction (HAB) should help to draw a firm conclusion about the abovementioned dual-task issue. It was hypothesized that jaw clenching has an effect on dynamic reactive balance, and this effect is not related to dual-task benefits, which would be indicated by the missing differences in dynamic reactive balance performance between the INT and JAW groups at T2.

## Methods

The study design comprised two measurement times (T1 and T2, separated by 1 week) and three groups (INT: intervention, JAW: jaw clenching, and HAB: habitual), whose details can be found in the following sections. The data of two groups (JAW and HAB) at T1 were partially presented in previously published studies (10, 36). An *a priori* power analysis was performed based on the study by Ringhof et al. (30) who analyzed the effects of submaximal jaw clenching on postural stability. The results revealed that 16 participants per group would be sufficient to reach a power of >0.8.

### Participants

A total of 48 physically active adults (20 women and 28 men; age: 23.2 ± 2.4 years; height: 1.74 ± 0.09 m; and body mass: 69.4 ± 10.4 kg) participated in this study. All participants gave written informed consent prior to the study, confirmed that they were participating in any kind of sports regularly at least three times per week, and were naive to the balance task instrument. They had no muscular or neurological diseases, showed no signs or symptoms of temporomandibular disorders [based on the Research Diagnostic Criteria for Temporomandibular Disorders (37)], and presented with full dentition (except for third molars) in neutral occlusion. The study was approved by the Ethics Committee of the Karlsruhe Institute of Technology.

### Study design

**Figure 1:**
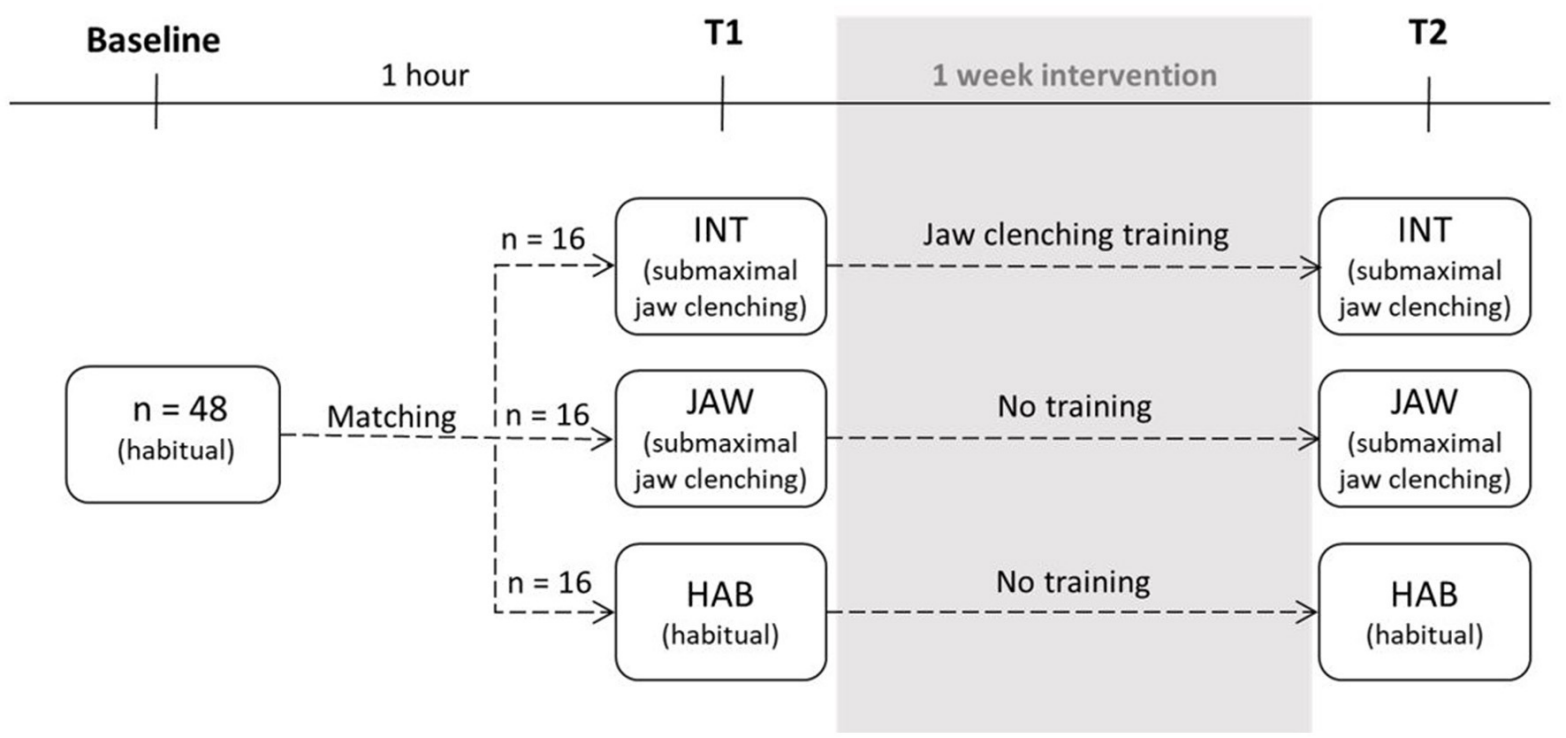
Study design (INT, intervention; JAW, jaw clenching; HAB, habitual; T1 and T2 are the measurement times).

To investigate whether the stabilizing effects of jaw clenching are merely a result of dual-task effects, the principal idea of our three-armed intervention study was that one of the groups, namely, INT, repeatedly practiced jaw clenching to make it a familiar and implicit task. The details of the three different groups (INT, JAW, and HAB) are shown in Figure 1.

Dynamic reactive balance performance was assessed using a commercially available oscillating platform (Posturomed, Haider-Bioswing, Weiden, Germany), which has previously been used to systematically investigate dynamic reactive balance performance after perturbations in many other studies (38–40). It is a rigid platform (12 kg, 60 × 60 cm) connected to a metal frame with eight steel springs (15 cm) of identical strength and can swing along the horizontal plane in all directions freely. A custom-made release system was used to apply mechanical perturbations in one of the four possible directions, back (*B*), front (*F*), left (*L*), and right (*R*), in a randomized order (10). Before the trials began, the participants were familiarized with the Posturomed with two trials without and two trials with perturbation. Afterward, a baseline measurement with a perturbation was conducted in the habitual stomatognathic motor condition to determine initial balance performance (10). Before each trial, participants were asked to stand on the platform on their dominant leg, hands at hips, eyes focusing on a fixed point at the eye level horizontally 4 m away from the center of the platform, and to compensate for the perturbation as quickly as possible. Their dominant leg was determined based on self-reports or, in case of uncertainty, by testing on the Posturomed (10, 41). In each trial, the platform was perturbed by the release system unpredictably in one of the four possible directions in a randomized order. The release system was used to release the platform from its maximum displaced position along the perturbation axis. After the perturbation, no external resistance forces were applied, and the participants had to dampen the perturbation by bringing the platform into its central position as soon as possible.

Both INT and JAW were jaw clenching groups and were instructed to clench their jaws during the balancing task. INT additionally trained in the jaw clenching task between T1 and T2, which were separated by 1 week. The purpose of this intervention was to make the novel jaw clenching task more automated, such that focused attention is reduced at T2. Groups were assigned considering the subjects' gender as well as their initial balance performance to ensure even distribution across the three groups. It was statistically confirmed that there were no baseline performance differences between the three groups (one-way ANOVA, *p* = 0.920).

During the balance task, INT and JAW were asked to clench their jaws with a force of 75 N. To familiarize themselves with this task, participants were trained for 5 min just before the measurements with a RehaBite^®^ (Plastyle GmbH, Uttenreuth, Germany), a medical training device consisting of liquid-filled plastic pads, to get used to applying this level of force (10, 42). During the measurements, the EMG activity of the masseter muscle corresponding to 75 N was used as a reference, and the participants in these two groups bit down on an Aqualizer^®^ intra-oral splint (medium volume; Dentrade International, Cologne, Germany). The HAB did not receive any instructions regarding the stomatognathic motor condition or an Aqualizer^®^. In the 1-week intervention phase between T1 and T2, INT trained three times a day for 10 min (10 repetitions of three sets, applying force for 10 s, stretching the jaw muscles, and resting for 10 s). For this purpose, the participants received a RehaBite^®^ and a diary to record the training sessions.

### Measurements

A total of 22 anthropometric measures were manually taken from each participant, and 42 reflective markers were placed on the participants' skin in accordance with the Advanced Lagrangian Solver in Kinetic Analysis modeling system [ALASKA, INSYS GmbH, Chemnitz, Germany (43)] to capture full body kinematics. Four reflective markers were attached to the upper surface of the Posturomed platform (10), and their displacements were captured using a 3D motion capture system (Vicon Motion Systems; Oxford Metrics Group, Oxford, UK; 10 Vantage V8 and 6 Vero V2.2 cameras; 200 Hz).

The activity of nine muscles [peroneus longus (PL), soleus (SOL), tibialis anterior (TA), rectus femoris (RF), semitendinosus (SM), rectus abdominis (AB), internal oblique (IO), erector spinae (ES), and masseter (MA)] was recorded using a wireless EMG system (Noraxon, Scottsdale, USA; 2,000 Hz) at the standing leg side. Before the measurements, the skin over the relevant muscles was shaved, abraded, and rinsed with alcohol. Bipolar Ag/AgCl surface electrodes (diameter 14 mm, center-to-center distance 20 mm; Noraxon Dual Electrodes, Noraxon, Scottsdale, USA) were attached in accordance with the European Recommendations for Surface EMG (44). Afterward, maximum voluntary contraction (MVC) tests were performed for normalization. At T1, the positions of EMG electrodes were marked with a temporary tattoo ink, so they could be placed in the same positions at T2.

A total of 12 valid trials (three per each of the four perturbation directions in a randomized order, each lasting 30 s) were recorded. Trials were invalid if participants did not apply enough force with their jaws (for INT and JAW), touched the ground with the non-standing foot, moved their standing foot, or released their hands from the hip. The success rate was high (i.e., only 1–2 invalid trials per participant) and did not differ between the groups. At T1 and T2, the same measurement process was followed.

### Data analysis

All data were recorded in Vicon Nexus 2.10 and processed with MATLAB R2021b (MathWorks). Kinematic data were filtered by a fourth-order Butterworth low-pass filter (10 Hz) and EMG data with a fourth-order Butterworth band-pass filter (10–500 Hz). The filtered EMG data were rectified and normalized to the MVC amplitudes (29). *R* and *L* directions were re-sorted into ipsilateral (*I*) and contralateral (*C*) according to the standing leg of the participants.

**Figure 2:**
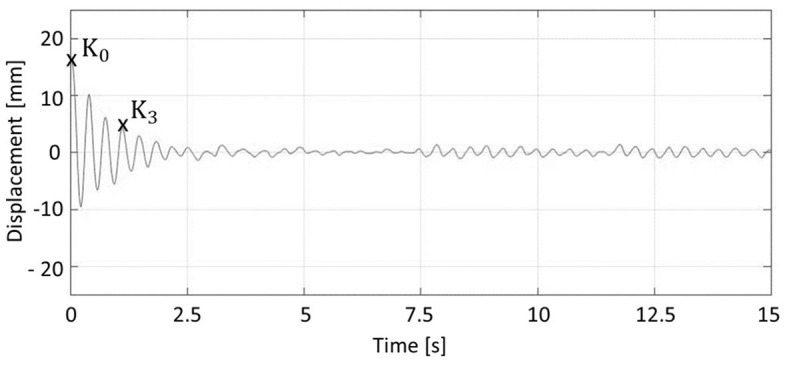
Calculation of damping ratio. The initial maximum displacement (K_0_) and the third positive amplitude (K_3_) were used for Eq.1.

To operationalize dynamic reactive balance performance, the damping ratio (10, 38) was calculated based on the movement of the Posturomed using the data of the markers attached to the platform (Equation 1, Figure 2). Larger damping ratio values represent better compensation of the perturbation and, therefore, better dynamic reactive balance and *vice versa*. With respect to the EMG data, three main latency responses were considered after the onset of perturbation: short (SLR, 30 to 60 ms), medium (MLR, 60 to 85 ms), and long (LLR, 85–120 ms) (40, 45). Two further time windows were considered: 100 ms before the onset of perturbation (PRE, −100–0 ms) and after the reflex phases until the end of the individual damping ratio (DRP, 120–1,136 ± 131 ms). Mean activities of the relevant muscles (directions *B*: PL and SOL; *F*: TA and AB; *I*: SM and IO, and *C*: RF and ES (46); MA for all directions) were calculated for the five phases, that is, PRE, SLR, MLR, LLR, and DRP.


(1) $$\begin{array}{c} \begin{array}{c} Damping\;ratio_{i}=\;\frac{\wedge }{\sqrt{\wedge_{i}^{2}+4\pi^{2}}}\;; \end{array}\\ \begin{array}{l} \wedge_{i}=\frac{1}{3}\ln\frac{K_{0}}{K_{i}}\;,\;K_{i}\;:\;i^{th}positive\;amplitude.\;(Equation\;1) \end{array} \end{array}$$

The marker trajectories in 3D were used to estimate the CoM trajectories with the full-body Dynamicus model [ALASKA, INSYS GmbH, Chemnitz, Germany (43)]. The COM displacement (47) was calculated as the range of motion of CoM along the perturbation axis (RoM_CoM_AP_ for *B* and *F*, and RoM_CoM_ML_ for *I* and *C*). Furthermore, the three-dimensional velocity of the CoM (*V*_CoM_) (48) was calculated for each trial and averaged for the whole damping ratio time window (0 ms until the end of the individual damping ratio).

### Statistics

Statistical calculations were done using IBM SPSS Statistics 25.0 (IBM Corporation, Armonk, NY, United States). For all dependent parameters (damping ratio, RoM_CoM_AP_, RoM_CoM_ML_, *V*_CoM_, and mean muscle activities), the three trials within each of the four perturbation directions were averaged. The normality of the data distributions was confirmed by Kolmogorov–Smirnov tests. The statistical assumptions were met to perform the repeated measures ANOVA (rmANOVA). The four perturbation directions were analyzed separately (10) since it was suggested that the direction of surface translation influences the sensation, central processing, and output of the postural responses differently (49, 50). For each dependent parameter, direction, and phase, a rmANOVA was calculated with the factors group (INT, JAW, and HAB) and time (T1 and T2). The significance level was set *a priori* to a p-value of < 0.05. In case of significant differences, *post hoc* tests or *t*-tests were performed for pairwise comparisons. Partial eta-squared and Cohen's d were calculated to quantify the effect sizes for rmANOVA and *post hoc* tests, respectively [small effect: $\eta_{p}^{2}$ < 0.06, d < 0.50; medium effect: 0.06 < $\eta_{p}^{2}$ < 0.14, 0.5 < d < 0.8; large effect: $\eta_{p}^{2}$ > 0.14, d > 0.8; (51)]. The Bonferroni–Holm method was applied to correct the results for multiple comparisons (52).

## Results

### Dynamic reactive balance performance

**Figure 3:**
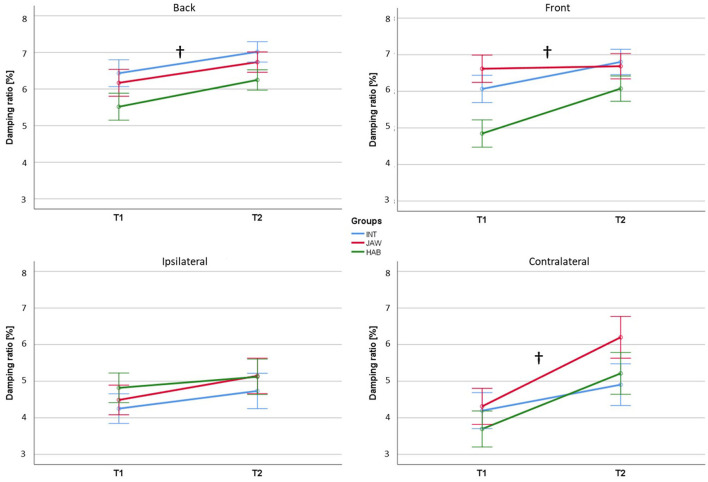
Damping ratio results. INT, intervention, JAW, jaw clenching, HAB, habitual. † signifies significant effects for the factor time. Significance level was set at *p* < 0.05.

The results regarding the damping ratio for the four directions are illustrated in Figure 3. For the factor time, rmANOVA results revealed significant improvements in the directions *B, F*, and *C* with high effect sizes (*B*: *p* = 0.042, $\eta_{p}^{2}$ = 0.168; *F*: *p* = 0.015, $\eta_{p}^{2}$ = 0.206; *C*: *p* < 0.001, $\eta_{p}^{2}$ = 0.356). However, there were no significant effects for the factor group as well as no interaction effects between the factors time and group. Accordingly, jaw clenching had no effect on dynamic reactive balance performance. In addition, the training of jaw clenching in the INT group did not show any effects on dynamic reactive balance performance. Independent of the groups, the dynamic reactive performance was better at T2 compared to T1.

### Center of mass kinematics

**Figure 4:**
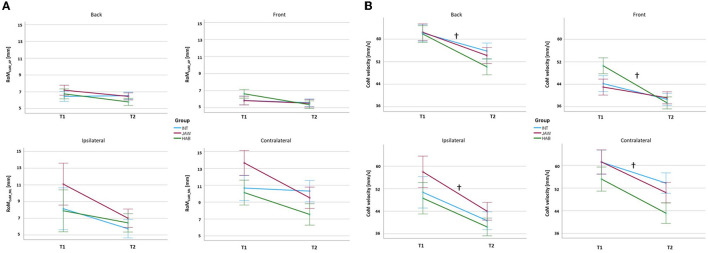
**(A)** RoM_CoM_AP_ and RoM_CoM_ML_ results **(B)** *V*_CoM_ results. INT, intervention; JAW, jaw clenching; HAB, habitual. † signifies significant effects for the factor time. The significance level was set at *p* < 0.05.

The RoM_CoM_AP_, RoM_CoM_ML_, and *V*_CoM_ results for the four directions are represented in Figures 4A, B. RoM_CoM_AP_ and RoM_CoM_ML_ did not show any significant effects. *V*_CoM_ had significant differences for the factor time in the directions *B, F, I*, and *C* with high effect sizes (*B*: *p* < 0.001, $\eta_{p}^{2}$ = 0.869; *F*: *p* = 0.004, $\eta_{p}^{2}$ = 0.289; *I*: *p* = 0.027, $\eta_{p}^{2}$ = 0.230; and *C*: *p* = 0.037, $\eta_{p}^{2}$ = 0.220). No significant effects for the factor group as well as no interaction effects between the factors time and group were detected. The results revealed that jaw clenching or its training had no significant effects on the center of mass kinematics. Across the groups, the *V*_CoM_ decreased at T2.

### Jaw clenching task controlled by masseter activity

The mean activities of the muscle MA for each phase are shown in Table 1. MA showed significant effects in all of the five phases for the factor time with medium effect sizes (PRE: *p* < 0.001, $\eta_{p}^{2}$ = 0.133; SLR: *p* < 0.001, $\eta_{p}^{2}$ = 0.116; MLR: *p* < 0.001, $\eta_{p}^{2}$ = 0.106; LLR: *p* < 0.001, $\eta_{p}^{2}$ = 0.113; and DRP: *p* < 0.001, $\eta_{p}^{2}$ = 0.121) and for the factor group with high effect sizes (PRE: *p* < 0.001, $\eta_{p}^{2}$ = 0.362; SLR: *p* < 0.001, $\eta_{p}^{2}$ = 0.364; MLR: *p* < 0.001, $\eta_{p}^{2}$ = 0.356; LLR: *p* < 0.001, $\eta_{p}^{2}$ = 0.351; and DRP: *p* < 0.001, $\eta_{p}^{2}$ = 0.340). In two of the five phases, there were significant interaction effects of the factors group and time with medium effect sizes (PRE: *p* = 0.002, $\eta_{p}^{2}$ = 0.066; and SLR: *p* = 0.003, $\eta_{p}^{2}$ = 0.061). *Post hoc* results showed that HAB had significantly lower MA activity with high effect sizes in all of the five phases in comparison to INT (PRE: *p* < 0.001, d = 1.268; SLR: *p* < 0.001, d = 1.260; MLR: *p* < 0.001, d = 1.240; LLR: *p* < 0.001, d = 1.229; DRP: *p* < 0.001, d = 1.225) as well as in comparison to JAW (PRE: *p* < 0.001, d = 1.674; SLR: *p* < 0.001, d = 1.681; MLR: *p* < 0.001, d = 1.641; LLR: *p* < 0.001, d = 1.621; and DRP: *p* < 0.001, d = 1.599).

**Table 1:** Mean muscle activities for Masseter in the five phases for all perturbation directions.

**All directions**		**PRE**		**SLR**		**MLR**		**LLR**		**DRP**	
		**T1**	**T2**	**T1**	**T2**	**T1**	**T2**	**T1**	**T2**	**T1**	**T2**
	INT	**7.37** ±9.47^*^^†^#	**4.80** ±4.95^*^^†^#	**7.36** ±9.84^*^^†^#	**4.50** ±4.55^*^^†^#	**7.65** ±9.48^*^^†^	**4.76** ±4.95^*^^†^	**7.02** ±8.77^*^^†^	**4.85** ±5.29^*^^†^	**7.15** ±8.74^*^^†^	**4.65** ±4.94^*^^†^
Masseter	JAW	**5.10** ±3.25^*^^†^#	**5.36** ±3.14^*^^†^#	**5.19** ±3.33^*^^†^#	**5.38** ±3.37^*^^†^#	**5.09** ±3.09^*^^†^	**5.32** ±3.26^*^^†^	**5.12** ±3.29^*^^†^	**5.26** ±3.07^*^^†^	**4.88** ±2.96^*^^†^	**5.11** ±2.93^*^^†^
	HAB	**1.47** ±2.90^*^^†^#	**0.45** ±0.68^*^^†^#	**1.51** ±2.98^*^^†^#	**0.48** ±0.93^*^^†^#	**1.53** ±2.95^*^^†^	**0.42** ±0.69^*^^†^	**1.49** ±2.84^*^^†^	**0.44** ±0.72^*^^†^	**1.51** ±2.77^*^^†^	**0.54** ±0.99^*^^†^

Significant effects are highlighted in bold. ^†^signifies significant effects for the factor time, ^*^for the factor group, and # the interaction effects. The significance level was set at p < 0.05.

These results indicated, first, that the MA activity at T1 was higher than at T2 independent of the group. Second, the group HAB had significantly lower MA activity compared to both jaw clenching groups, INT and JAW, independent of the measurement time. Third, the reduction in MA activity level from T1 to T2 was partly higher for the jaw clenching group, INT, that trained for the task between two measurement times compared to JAW and HAB.

### Muscle activities in the critical phases for reflexes

The mean activities of the analyzed muscles for each phase are shown in Tables 2, 3 for anterio-posterior and medio-lateral perturbations, respectively. The significant effects are highlighted in the tables. The corresponding *p*-values and effect sizes are reported in the following paragraphs.

**Table 2:** Mean muscle activities for perturbation-relevant muscles in the five phases for anterio-posterior perturbations.

**Back**		**PRE**		**SLR**		**MLR**		**LLR**		**DRP**	
		**T1**	**T2**	**T1**	**T2**	**T1**	**T2**	**T1**	**T2**	**T1**	**T2**
Peroneus longus	INT	14.41 ± 10.92	13.74 ± 9.68	16.82 ± 16.77	17.68 ± 17.08	17.17 ± 12.68	18.54 ± 18.13	14.81 ± 10.70	19.46 ± 15.65	22.63 ± 11.40	22.56 ± 14.41
	JAW	21.08 ± 11.67	12.75 ± 6.80	22.94 ± 15.97	16.16 ± 11.00	21.04 ± 11.88	14.39 ± 9.14	20.13 ± 11.78	17.16 ± 12.68	26.51 ± 10.56	21.06 ± 10.22
	HAB	37.55 ± 74.72	19.41 ± 18.60	26.26 ± 35.13	20.87 ± 16.98	44.48 ± 99.47	23.17 ± 30.02	45.94 ± 103.20	22.14 ± 23.77	37.21 ± 41.44	25.42 ± 18.58
Soleus	INT	19.62 ± 12.34	15.31 ± 5.02	**21.22** **±11.71#**	**13.75** **±5.59#**	20.60 ± 24.14	14.83 ± 11.05	21.81 ± 19.00	18.38 ± 12.32	26.36 ± 13.56	24.26 ± 15.23
	JAW	14.63 ± 6.71	18.32 ± 7.38	**12.81** **±6.19#**	**18.32** **±9.54#**	15.43 ± 12.10	14.31 ± 7.70	18.42 ± 12.82	16.77 ± 10.09	21.77 ± 10.08	23.57 ± 11.87
	HAB	16.23 ± 10.66	12.95 ± 7.97	**16.95** **±13.18#**	**11.41** **±5.91#**	15.54 ± 16.53	10.19 ± 4.82	13.01 ± 9.51	12.92 ± 7.06	20.83 ± 11.57	19.56 ± 9.38
**Front**		**PRE**		**SLR**		**MLR**		**LLR**		**DRP**	
		**T1**	**T2**	**T1**	**T2**	**T1**	**T2**	**T1**	**T2**	**T1**	**T2**
Tibialis anterior	INT	**11.96** **±13.00**^**†**^	**5.93** **±7.86**^**†**^	**12.68** **±15.92**^**†**^	**5.30** **±5.83**^**†**^	**11.79** ±11.29^*^	**4.42** ±3.71^*^	9.57 ± 8.28	6.28 ± 5.06	**14.23** **±5.83**^**†**^	**10.34** **±6.42**^**†**^
	JAW	**9.63** **±5.85**^**†**^	**6.05** **±2.86**^**†**^	**10.59** **±9.50**^**†**^	**5.17** **±3.02**^**†**^	**10.86** ±7.11^*^	**5.38** ±2.94^*^	9.60 ± 6.94	5.82 ± 5.20	**15.40** **±8.23**^**†**^	**9.66** **±5.14**^**†**^
	HAB	**7.09** **±5.28**^**†**^	**5.79** **±2.78**^**†**^	**5.50** **±4.01**^**†**^	**5.78** **±3.81**^**†**^	**6.39** ±4.15^*^	**6.50** ±5.71^*^	6.19 ± 3.92	7.03 ± 4.45	**13.41** **±7.28**^**†**^	**11.49** **±5.93**^**†**^
Rectus abdominis	INT	1.45 ± 2.13	0.91 ± 1.38	1.46 ± 2.51	0.94 ± 1.46	1.43 ± 2.05	0.84 ± 1.15	1.37 ± 1.70	0.81 ± 1.24	1.45 ± 1.59	1.08 ± 1.63
	JAW	0.86 ± 0.57	0.90 ± 0.68	0.86 ± 0.77	0.81 ± 0.74	0.75 ± 0.43	0.82 ± 0.61	0.96 ± 0.75	0.86 ± 0.90	1.08 ± 0.72	0.91 ± 0.68
	HAB	1.14 ± 1.12	1.27 ± 1.02	1.16 ± 1.12	1.07 ± 0.96	1.08 ± 1.07	1.18 ± 0.95	0.90 ± 0.76	1.31 ± 1.04	1.43 ± 1.17	1.26 ± 0.85

Significant effects are highlighted in bold. ^†^signifies significant effects for the factor time, ^*^for the factor group, and # the interaction effects. The significance level was set at p < 0.05.

**Table 3:** Mean muscle activities for perturbation-relevant muscles in the five phases for medio-lateral perturbations.

**Ipsilateral**		**PRE**		**SLR**		**MLR**		**LLR**		**DRP**	
		**T1**	**T2**	**T1**	**T2**	**T1**	**T2**	**T1**	**T2**	**T1**	**T2**
Semitendinosus	INT	5.04 ± 5.50	4.11 ± 6.86	7.17 ± 9.62	3.86 ± 5.55	6.28 ± 9.69	4.48 ± 7.11	6.12 ± 8.20	4.80 ± 8.75	7.95 ± 7.73	6.53 ± 8.51
	JAW	3.88 ± 3.41	3.11 ± 2.39	3.48 ± 3.53	3.04 ± 3.05	4.46 ± 4.06	3.65 ± 3.13	4.10 ± 3.23	3.76 ± 3.19	5.85 ± 4.86	4.06 ± 2.50
	HAB	4.99 ± 4.43	3.87 ± 5.10	4.65 ± 4.00	4.00 ± 5.46	4.69 ± 4.35	4.57 ± 5.82	5.20 ± 4.87	4.30 ± 5.79	6.75 ± 5.87	5.66 ± 6.99
Internal oblique	INT	2.85 ± 2.66	1.95 ± 1.77	3.33 ± 3.26	2.58 ± 1.96	2.95 ± 3.11	2.66 ± 2.42	3.16 ± 3.47	2.75 ± 2.89	3.72 ± 3.06	3.28 ± 2.34
	JAW	2.68 ± 1.54	1.87 ± 1.61	2.69 ± 1.69	2.52 ± 2.20	2.54 ± 1.29	2.65 ± 2.64	2.64 ± 1.39	2.80 ± 2.47	3.50 ± 1.55	3.30 ± 2.13
	HAB	2.21 ± 1.19	2.08 ± 1.41	2.24 ± 1.31	1.82 ± 1.46	2.22 ± 1.30	1.88 ± 1.17	2.09 ± 1.02	2.01 ± 1.31	3.91 ± 3.13	2.85 ± 2.09
**Contralateral**		**PRE**		**SLR**		**MLR**		**LLR**		**DRP**	
		**T1**	**T2**	**T1**	**T2**	**T1**	**T2**	**T1**	**T2**	**T1**	**T2**
Rectus femoris	INT	2.27 ± 4.32	3.05 ± 3.04	3.78 ± 2.77	3.29 ± 3.28	4.81 ± 8.34	3.13 ± 3.11	4.55 ± 7.35	3.33 ± 3.57	5.31 ± 3.93	5.23 ± 5.59
	JAW	3.82 ± 2.27	3.37 ± 3.30	5.42 ± 4.40	3.27 ± 3.06	3.61 ± 2.55	3.63 ± 3.76	4.74 ± 2.91	3.89 ± 4.22	6.23 ± 3.87	5.02 ± 3.88
	HAB	2.71 ± 2.69	2.62 ± 1.96	2.51 ± 1.98	2.60 ± 2.19	2.88 ± 2.91	2.45 ± 1.81	2.87 ± 2.62	2.60 ± 1.60	4.78 ± 4.87	4.23 ± 2.67
Erector spinae	INT	5.13 ± 3.17	7.00 ± 6.42	5.24 ± 3.06	5.74 ± 3.94	6.84 ± 5.59	7.25 ± 7.71	7.20 ± 6.47	6.80 ± 6.92	7.85 ± 5.10	10.22 ± 8.10
	JAW	4.57 ± 4.93	4.44 ± 3.56	4.06 ± 4.32	4.09 ± 3.08	4.73 ± 4.75	4.50 ± 4.05	4.84 ± 3.76	4.76 ± 4.73	8.13 ± 7.91	6.16 ± 4.51
	HAB	4.26 ± 3.45	4.79 ± 3.42	4.74 ± 4.01	4.58 ± 3.84	5.69 ± 7.52	4.16 ± 2.96	4.32 ± 3.30	4.41 ± 3.37	8.15 ± 7.11	6.98 ± 5.17

For direction *B*, the muscle SOL showed significant interaction effects between the factors time and group with high effect sizes in SLR (*p* = 0.002, $\eta_{p}^{2}$ = 0.240). At T2, the group JAW had an increased level of SOL activity compared to T1, whereas the other two groups had a decreased level. For the direction *F*, the muscle TA showed significant effects with high effect sizes for the factor time in three of the five phases (PRE: *p* < 0.001, $\eta_{p}^{2}$ = 0.269; SLR: *p* = 0.003, $\eta_{p}^{2}$ = 0.177; and DRP: *p* < 0.001, $\eta_{p}^{2}$ = 0.333) as well as for the factor group in one phase (MLR: *p* < 0.001, $\eta_{p}^{2}$ = 0.306). Across all groups, the level of TA activity was decreased at T2 compared to T1 in PRE, SLR, and DPR. Furthermore, across the measurement times, the JAW and INT groups had a higher level of TA activity compared to HAB in MLR. The *post hoc t*-test results revealed that these differences were valid at T1 but not at T2. For directions *C* and *I*, no significant effects were found.

In summary, the results showed that the reflex activity changes were limited to anterior–posterior directions (*B* and *F*). In the case of backward acceleration of the platform, the JAW group showed increases in SOL activity at T2, whereas the other two groups revealed decreases. In the case of forward acceleration of the platform, the TA activity was lower at T2 compared with T1 in three reflex phases, independent of the groups. Furthermore, the two jaw clenching groups (JAW and INT) had higher TA activity compared to HAB in the MLR phase at T1.

## Discussion

This study aimed to investigate the effects of jaw clenching training on a dynamic reactive balance task performance after 1 week of jaw clenching training. It was hypothesized that jaw clenching has stabilizing effects resulting in better dynamic reactive balance performance, and these effects persist at T2 after the intervention. This would mean that these improvements are not a result of the dual-task effect but are specifically associated with jaw clenching. The results indicated that neither jaw clenching nor its automation through training resulted in significant dynamic reactive balance performance differences. However, independent of the groups, the dynamic reactive balance performance was better at T2 compared to T1. As there was not any deliberate balance training in the intervention phase, this result is indicative of high learning effects. Furthermore, jaw clenching may lead to some changes in reflex activities, but they are limited to anterior–posterior perturbation of the platform.

### Effects of jaw clenching on dynamic reactive balance performance and CoM kinematics

Dynamic reactive balance performance was operationalized by the damping ratio as in other studies (10, 38). In addition, the RoM of CoM along the perturbation axis, as well as *V*_CoM_, were calculated. In all of the directions, no significant effects due to jaw clenching were observed. Previous studies showed that jaw clenching may affect balance performance under static steady-state conditions (12, 29, 30, 53) as well as under dynamic conditions (10, 14). However, the nature of these effects is still unknown and could be associated with the dual-task situation. To the best of our knowledge, research so far has not addressed this point explicitly. This study investigated the effects of jaw clenching on dynamic reactive balance performance after 1 week of jaw clenching training to determine whether the effects are a result of the general dual-task situation or specifically due to the neurophysiological effects of jaw clenching. At T1 and T2, both INT and JAW groups were instructed to do the same dual task. These two groups differed only in the intervention: INT trained in the jaw clenching task, whereas JAW did not. It was assumed that after 1 week of training (18 training sessions with 10 min of practice), the participants of INT would be able to fulfill the jaw clenching task in an automated manner. Therefore, it was hypothesized that the INT group would have reduced focused attention on the secondary jaw clenching task (31) and, therefore, a worse balance performance than JAW at T2. However, the results did not reveal any significant performance differences between the groups. Based on this, it can be concluded that the jaw clenching task did not have any observable effects on dynamic reactive balance performance, which was operationalized by the damping ratio and CoM kinematics. Furthermore, its automation also did not result in any significant changes. On the other hand, another explanation might be that the response of the motor system to the complexity of the present balance task possibly masked the effects of jaw clenching, which were identified in previous experiments with static balance tasks (29, 30). In addition, in a previous study by Tardieu et al. (54), the effects of dental occlusion on postural control were investigated both in eyes open and closed conditions. They reported that the sensory information associated with dental occlusion becomes more important when the other sensory cues become scarce (e.g., eyes closed). Based on this, it can be suggested that a jaw clenching task might potentially be beneficial once sensory information from other sources reduces. Nevertheless, in this study, the balance task was performed with open eyes since the Posturomed task was too difficult to be handled with closed eyes.

### High learning effects even without training between sessions

In three of four directions (*B, F*, and *C*), dynamic reactive balance performance was improved at T2 even though the participants did not perform any balance training between T1 and T2. Furthermore, in all directions, *V*_CoM_ decreased significantly at T2, whereas the RoM_CoM_AP_ and RoM_CoM_ML_ were not affected. It should be noted that the participants performed familiarization trials before the real measurements as in similar studies (40, 55). Furthermore, within the individual measurement session, there were no systematic performance improvements in terms of dynamic reactive balance. These results indicate that learning effects occurred without deliberate balance training for this specific task. Subsequently, the question arose whether the learning effects were so large that they outweighed the possible effects of jaw clenching. With this study design, this question cannot be answered, and further studies are needed. From the findings of this study, it can be concluded that the balance task used here shows high learning effects and is rather unsuitable for studies in which low intervention effects on balance performance are expected. In the present case as well as in similar cases, care should therefore be taken to select a balance task that shows only low learning effects or a longer intervention period should be scheduled between T1 and T2 to mitigate the unwanted learning effects.

The results also revealed that the velocity of the CoM changed, but its RoM in the perturbation direction did not change at T2. This may be explained by the decreased CoM movement in case of the better damping of the platform by the participants since the CoM is one of the controlled variables as suggested in postural studies (56, 57). On the other hand, RoM_CoM_AP_ and RoM_CoM_ML_ depended for the most part on the initial maximum displacement of the platform, which was identical at T1 and T2. Therefore, the RoM did not change at T2.

### Changed muscular activity levels at T2

The results regarding muscle activities in reflex phases revealed that in the case of the backward perturbation of the platform (*B*), the SOL activity of JAW increased, whereas that of the other two groups decreased at T2 in the SLR phase. It is important to add that SOL is one of the most important muscles that help to restore equilibrium in response to posterior translations (46). This result may be interpreted as a difference between INT and JAW groups, and it can be suggested that the jaw clenching task resulted in increased muscle activity in SOL at T2, but these effects were not visible when the jaw clenching task became an implicit task and therefore lost its novelty (e.g., for the group INT). In addition, in the case of forward acceleration of the platform, TA activity of both jaw clenching groups (INT and JAW) was higher overall compared to that of HAB in the MLR phase at T1. This finding contradicts the initial hypothesis that the jaw clenching task results in changes in reflex activities, and these effects persist after 1 week of jaw clenching training. Nevertheless, these results are only limited to this perturbation direction and to this specific reflex phase. Furthermore, these changes did not cause any effects on dynamic reactive balance performance (i.e., damping ratio results).

In response to anterior surface translations, TA contracts to counteract the torques at the ankle and, therefore, helps to restore equilibrium (46). The TA activity decreased at T2 in three phases (PRE, SLR, and DRP) across the groups parallel to dynamic balance performance improvements. These results indicate that in the case of forward acceleration of the platform, better performance at T2 is possibly related to a decreased TA activity. In general, significant changes were detected only for the anterior–posterior perturbation directions. Based on these results, it can be suggested that the jaw clenching task may result in changed muscle activity patterns, as observed with the alterations in certain muscle activities in the reflex phases, but changes seem to be direction-dependent as well as muscle dependent. This task specificity can be explained by the different postural responses to different perturbation directions (49, 50, 58).

Furthermore, it should be noted that the muscle activity changes, and the dynamic balance performance differences did not show a common pattern for all directions (e.g., no changes in muscle activity levels in perturbation direction *C*, despite the improvements in dynamic reactive balance performance at T2). This may also possibly have been caused by the selection of the posture relevant muscles. Posture and its control are the product of inter-muscular coordination patterns. Determining the activity of individual muscles might be the limiting factor in the analysis presented here. In light of these aspects, the question arises whether mean muscle activities for the critical phases were sensitive enough to reveal changes on a muscular level. Nevertheless, these parameters were used in similar studies [e.g., iEMG in Freyler et al. (40) and Pfusterschmied et al. (39)]. In the present study, mean muscle activity was preferred since DRP was not the same length for each trial or participant. It was expected that an increased level of reflex activities would be manifested by an increased level of muscle activities (59). However, potentially jaw clenching effects are seen less in a changed level of individual muscle activities and more in a changed interplay of different muscles. Therefore, in future studies, the coordination of different muscles should be analyzed in addition to the analysis of the activity of individual muscles. Coordination models, such as muscle synergies, are particularly suitable for this purpose (60, 61).

### Jaw clenching task controlled by masseter activity

The EMG results indicate that the activity of the MA was higher for the groups, INT and JAW compared to HAB. This suggests that the majority of the subjects in HAB, who did not receive instructions regarding the activity of the stomatognathic system, had their jaws in the physiologically expected resting position (lips closed, teeth out of contact). It should be noted that the participants of JAW and INT trained immediately before starting the balancing task measurements with the Rehabite^®^ device so they can apply a force at a level of 75 N consistently without feedback. The higher reduction in MA activity between T1 and T2 in the INT group compared with the other groups can be attributed to the training during the intervention phase. Similar effects were also shown in a previous study (62), in which short-term force-controlled biting on a hydrostatic system caused long-term training effects.

A force of 75 N is easy to achieve for the stomatognathic system as normal masticatory activities are in the range of this force level. The RehaBite^®^-training in the group INT between T1 and T2 was used to turn a novel, unfamiliar task (biting on a hydrostatic system is not part of the common functional repertoire of the stomatognathic system) into an implicit behavior so that it would not require additional attention during the balancing task. Therefore, RehaBite^®^ training between T1 and T2 in INT was not used to train the masticatory muscles but to address a potential dual-task effect during the balance task. It should also be noted that the jaw clenching task in this study is a different stomatognathic activity than the daily chewing activity occurring when eating (62). During the submaximum jaw clenching task, a force of 75 N was applied continuously, whereas, during chewing, an alternating force is applied. Based on this, it can be assumed that the deliberate jaw clenching task was novel to the participants at the first measurements. Furthermore, it was also shown that the chewing task had no significant effects on body sway reduction during upright standing, whereas the feedback-controlled jaw clenching task had significant effects (53). This also supports that the submaximum jaw clenching and the chewing tasks are not the same task, and they may lead to different neurophysiological effects.

### Limitations

This study has some limitations. First, even though the participants did not train for the balance task, learning effects occurred in three of the four directions independent of the group. These high learning effects may have outweighed the potential effects of jaw clenching. For future studies, more care should be taken to minimize possible learning effects. Second, all the participants were physically active and healthy adults, therefore potentially good at balancing. The same results may not be seen in groups with compromised postural control such as the elderly (63) or people with neurological disorders (64). In future studies, the participants with poorer postural control might reveal the effects of jaw clenching. Third, the onset of the reflex phases was defined based on Posturomed movement but not on muscle activity peaks (45) or ankle movements since there were no clear peaks in the EMG or kinematics data. Finally, the group HAB did not receive any instructions regarding stomatognathic activities. Self-administrative questionnaires regarding the clenching habit would have been useful to collect habitual status.

## Conclusion

This study investigated the effects of jaw clenching on dynamic reactive balance task performance after 1 week of jaw clenching training to examine whether the effects are a result of a dual-task situation. Both jaw clenching and automation of the jaw clenching task seemed not to have any observable effects on dynamic reactive balance performance, but jaw clenching seemed to be related to some changes in reflex activities. However, these effects were limited to anterior–posterior perturbations. Further studies containing other balance tasks with less learning effects as well as with longer intervention periods are needed. Analysis of muscle coordination, as well as other experimental designs with reduced sensory information from other sources (e.g., closed eyes), may also help to reveal jaw clenching effects.

## Data availability statement

The raw data supporting the conclusions of this article will be made available by the authors, without undue reservation.

## Ethics statement

The studies involving human participants were reviewed and approved by Ethics Committee of the Karlsruhe Institute of Technology. The participants provided their written informed consent to participate in this study.

## Author contributions

CF and LK conducted the experiment. CF carried out data analysis and took the lead in writing the manuscript. All authors were involved in the interpretation and discussion of the results, provided critical feedback, contributed to the article, and approved the submitted version.
